# Exosomal *NOX1* promotes tumor-associated macrophage M2 polarization-mediated cancer progression by stimulating ROS production in cervical cancer: a preliminary study

**DOI:** 10.1186/s40001-023-01246-9

**Published:** 2023-09-07

**Authors:** Liying Gu, Chunyang Feng, Meng Li, Zubei Hong, Wen Di, Lihua Qiu

**Affiliations:** 1 Department of Obstetrics and Gynecology, Ren Ji Hospital, Shanghai Jiao Tong University School of Medicine, Shanghai, China; 2 Shanghai Key Laboratory of Gynecologic Oncology, Ren Ji Hospital, Shanghai Jiao Tong University School of Medicine, Shanghai, China; 3 State Key Laboratory of Oncogenes and Related Genes, Shanghai Cancer Institute, Ren Ji Hospital, Shanghai Jiao Tong University School of Medicine, Shanghai, China; 4 Department of Oral Mucosal Diseases, Shanghai Ninth People’s Hospital, Shanghai Jiao Tong University School of Medicine, College of Stomatology, Shanghai Jiao Tong University, Shanghai, China; 5 National Center for Stomatology, National Clinical Research Center for Oral Diseases, Shanghai Key Laboratory of Stomatology, Shanghai, China

**Keywords:** Exosome, Tumor-associated macrophage, M2 polarization, *NOX1*, ROS, Cervical cancer

## Abstract

**Background:**

Cervical cancer the fourth most frequently diagnosed cancer and the fourth leading cause of cancer death in women, with an estimated 604,000 new cases and 342,000 deaths worldwide in 2020 for high rates of recurrence and metastasis. Identification of novel targets could aid in the prediction and treatment of cervical cancer. NADPH oxidase 1 (NOX1) gene-mediated production of reactive oxygen species (ROS) could induce migration and invasion of cervical cancer cells. Tumor-associated macrophages (TAMs) play important roles in cervical cancer. Tumor cell-derived exosomes mediate signal transduction between the tumor and tumor microenvironment. Elucidation of the mechanisms of NOX1-carrying exosomes involved in the regulation of TAMs may provide valuable insights into the progression of cervical cancer.

**Methods:**

Uniformly standardized mRNA data of pan-carcinoma from the UCSC database were downloaded. Expression of *NOX1* in tumor and adjacent normal tissues for each tumor type was calculated using R language software and significant differences were analyzed. SNP data set were downloaded for all TCGA samples processed using MuTect2 software from GDC. Cell experiment and animal tumor formation experiment were used to evaluate whether exosomal *NOX1* stimulating ROS production to promote M2 polarization of TAM in cervical cancer.

**Results:**

*NOX1* is highly expressed with a low mutational frequency in pan-carcinoma. Upregulation of *NOX1* may be associated with infiltration of M2-type macrophages in cervical cancer tissues, and *NOX1* promotes malignant features of cervical cancer cells by stimulating ROS production. Exosomal *NOX1* promotes M2 polarization of by stimulating ROS production. Exosomal *NOX1* enhances progression of cervical cancer and M2 polarization in vivo by stimulating ROS production.

**Conclusion:**

Exosomal *NOX1* promotes TAM M2 polarization-mediated cancer progression through stimulating ROS production in cervical cancer.

**Supplementary Information:**

The online version contains supplementary material available at 10.1186/s40001-023-01246-9.

## Introduction

Cervical cancer, a heterogeneous tumor type, is the third most commonly diagnosed cancer in women worldwide. While screening programs and HPV vaccinations have had a successful impact in reducing the incidence of cervical cancer, over 13,000 cases continue to be diagnosed each year in the United States.[1]. From Global Cancer Statistics 2020, the data showed in 2020, there were 604,127 new cases of cervical cancer worldwide, of which 109,741 were in China. The number of deaths from cervical cancer worldwide in 2020 was 341,831, of which 59,060 were in China [2]. Surgery, chemotherapy, radiotherapy, and immunotherapy are the common modalities of treatment, but high rates of recurrence and metastasis remain the main causes of death [3, 4]. Identification of novel targets that could aid in the prediction and treatment of cervical cancer thus remains an urgent unmet clinical need.

NADPH oxidase 1 (*NOX1*), located on the X chromosome, is reported to be involved in a variety of pathogenic mechanisms including vascular disease, tumor angiogenesis, and fibrosis [5]. Recently, overexpression of DEAD-box helicase 19A (DDX19A) was reported to increase NOX-1-mediated production of reactive oxygen species (ROS) to induce migration and invasion of cervical squamous cell carcinoma cells. Additionally, high expression of *NOX1* was associated with significantly decreased overall patient survival [6]. However, expression patterns of the *NOX1* gene in cervical cancer and adjacent tissues and its tumor-promoting mechanisms remain to be established.

Exosomes, a type of extracellular vesicles, are released by almost all eukaryotic cells, including tumor cells. Tumor cell-derived exosomes mediate signal transduction between the tumor and surrounding non-tumor cells. This intercellular communication actively contributes to remodeling of the tumor microenvironment (TME) to facilitate tumor growth, invasion, and metastasis [7]. The importance of the TME in dynamic regulation of cancer progression is widely accepted [8]. Tumor-associated macrophages (TAMs) in the TME play important roles in the genesis, development, and prognosis of cervical cancer [9]. Earlier studies indicate that TAMs are redefined into anti-tumor M1-like and pro-tumor M2-like TAMs during tumor development [10]. The relationship between NOX1 and exosomes has also been reported in the published literature, Vytautas Žėkas et al. found Oxidative Properties of Blood-Derived Extracellular Vesicles in 15 Patients After Myocardial Infarction [11]. Renato et al. found platelet-derived extracellular vesicles express NADPH oxidase-1 (*NOX1*), generate superoxide, and modulate platelet function [12]. These studies demonstrate that NOX1 can indeed act through the exosome pathway.

Elucidation of whether *NOX1*-carrying exosomes are involved in the regulation of TAMs may therefore provide valuable insights into the mechanisms associated with progression of cervical cancer.

In this study, we investigated the expression patterns of *NOX1* in cervical cancer. Our collective findings suggest that *NOX1*-carrying exosomes promote the malignant features of cervical cancer by regulating TAMs via ROS production, providing a theoretical basis and novel targets for effective clinical treatment of cervical cancer.

## Materials and methods

### Bioinformatics analysis

In terms of data sources for bioinformatics analysis, we downloaded uniformly standardized mRNA data of pan-carcinoma from the UCSC database (https://xenabrowser.net/). Expression of *NOX1* in tumor and adjacent normal tissues for each tumor type (Additional file 2: Table S1) was calculated using R language software (version 3.6.4) and significant differences analyzed using unpaired Wilcoxon Rank Sum and Signed Rank Tests. We downloaded the simple nucleotide variation data set for all TCGA samples processed using MuTect2 software from GDC (https://portal.gdc.cancer.gov/). The R language software package “maftools” (version 2.2.10) was used for mapping.

### Clinical samples

Ten pairs of cervical cancer and adjacent tissues were collected between January 2020 and June 2020 from patients in the Department of Obstetrics and Gynecology, Renji Hospital, Shanghai Jiao Tong University School of Medicine(Additional file 2: Table S2). None of the patients had undergone chemotherapy or radiotherapy before surgery. Histologic examinations were performed by two pathologists from the Department of Pathology at our hospital based on WHO criteria. This study was approved by the Institutional Review Board of Shanghai Renji Hospital (Approval number: KY2020-018). Written informed consent was obtained from all patients.

### Cell culture

The cervical cancer cell line, HeLa (ZQ0068, Shanghai Zhongqiao Xinzhou Biotechnology Co., Ltd., China), and THP-1 cells (ZQ0086) were cultured in DMEM supplemented with 10% fetal bovine serum and 1% penicillin and streptomycin in a humidified incubator at 37 ℃ and 5% CO_2_.

### Macrophage induction

THP-1 cells were treated with phorbol 12-myristate 13-acetate (PMA, 100 ng/mL; P1585, Sigma, USA) for 48 h. CD14 monoclonal antibody (APC) (17-0149-41, eBioscience, USA) or PE anti-mouse/human CD11b (101207, BioLegend, USA) fluorescent antibody was added to the cell suspension and incubated for 30 min away from light. The proportions of CD14- and CD11b-positive cells were detected via flow cytometry (CytoFLEX, Beckman, USA).

### Construction of stable cell lines

pCDH-CMV-MCS-EF1-copGFP-T2A-Puro as the negative control (NC) and pCDH-CMV-MCS-EF1-*NOX1*-Puro were synthesized by RiboBio (Guangzhou, China). HeLa cells were inoculated into 96-well plates for 24 h before the experiment. At a cell confluence of ~ 80%, different concentrations of puromycin (0, 0.1, 0.25, 0.5, 0.75, 1, 2, 5 and 10 µg/mL) were added. The screening concentration of puromycin resistance was 2 µg/mL. Next, HeLa cells were inoculated into 6-well plates and cultured with 5 µg/mL polybrene and lentivirus for 48 h. Cells co-cultured with 20 µM ROS inhibitors (acetylcysteine (Ace), MCE, HY-B0215) were used for subsequent experiments.

### Real-time fluorescence quantitative PCR (RT-PCR)

Total RNA from tissues or cells was extracted using TRIzol (1559601; Ambion, USA) and reverse transcribed into cDNA using a PrimeScript™ RT reagent Kit (RR037Q, Takara, Japan). qPCR was further performed using TB Green^®^ Fast qPCR Mix (RR430A, Takara) and 2^–ΔΔCT^ was applied to calculate relative gene expression using GAPDH as the internal reference. All primer sequences are presented in Additional file 2: Table S3.

### Immunohistochemical (IHC) analysis

Paraffin-embedded samples were cut into 3 μm sections, dewaxed with xylene, and rehydrated with graded ethanol. For antigen recovery, sections were heated at 97 °C for 20 min, digested briefly via proteolysis, blocked with peroxidase, and incubated overnight with *NOX1* polyclonal antibody (1:200, 17772-1-AP, Proteintech, USA) at 4 °C using HRP/Fab polymer conjugates (A0208, Beyotime, China) as secondary antibodies. Finally, diaminobenzidine substrate was used for staining and hematoxylin was used for re-staining. Slices were observed under a fluorescence microscope (ECLIPSE Ts2, Nikon, Japan).

### Immunofluorescence staining

Immunofluorescence analysis was performed as described in previous report. Expression of CD68, iNOS, and CD163 in tissues and cells was examined via overnight incubation with CD68 antibody (1:100; AB201340, Abcam, USA), CD163 antibody (1:400; AB182422, Abcam) and iNOS antibody (1:200; Nb300-605, Novus Biologicals, USA). Samples were subsequently incubated with secondary HRP-labeled goat anti-rabbit IgG (A0208, Beyotime) or HRP-labeled goat anti-mouse IgG (A0216, Beyotime). Nuclei were stained with DAPI solution (E607303, Sangon Biotech, China) and fluorescence images observed using confocal laser fluorescence microscopy (ECLIPSE Ts2, Nikon, Japan).

### Exosome isolation

HeLa cells were cultured in DMEM supplemented with 10% exosome-free fetal bovine serum (SBI, CA, USA) for 48 h, and exosomes from cell supernatants were purified via differential super-centrifugation, 300*g*, 10 min, 2000*g*, 10 min, 10000*g*, 30 min, and 100000*g*, 75 min.

### Transmission electron microscopy (TEM)

Exosomes were re-suspended in PBS, immobilized in 30 μL of 2% paraformaldehyde, and adsorbed on a light-discharging copper grid. The copper grid attached to exosomes was suspended in 3% glutaraldehyde droplets, fixed in 0.1 M phosphate buffer of 1% glutaraldehyde (pH 7.4), and suspended on 4% uranyl acetate droplets for exosomal staining. Transmission electron microscopy (Tecnai G2 Spirit BioTwin, FEI, USA) was employed for image acquisition.

### Nanoparticle tracking analysis (NTA)

Exosome suspensions were collected after adding 10 μL of 10% glycine to 10 μL vesicles in 90 μL buffer, diluted with PBS to a final ratio of 1:200, and lysed with mammalian protein extraction reagent (Millipore, Billerica, MA, USA). Exosome diameters (nm) were calculated using a Nano Particle tracking analyzer (ZetaVIEW S/N 17-310, Particle Metrix, Germany).

### Immunofluorescence analysis of exosome uptake

Following induction of THP-1 cells into M0 macrophages, co-culture was performed with 10 µg/mL HeLa-NC (blank exosomes) or HeLa-OE (*NOX1*-overexpressing exosomes) for 0.5, 2, and 12 h, following which cells were fixed with 4% paraformaldehyde at room temperature for 30 min. FITC-labeled phalloidin working solution (250 µL) prepared with 1% BSA (100 nM, 40735ES75; Yeasen, China) was added to each well and incubated at room temperature in the dark for 30 min. Following the addition of anti-fluorescence quenching agent (E675011; Sangon Biotech, China), cells were visualized under a microscope (ECLIPSE Ts2, Nikon, Japan).

### Western blot (WB)

Total protein obtained using RIPA lysis solution (Beyotime, China) was transferred to fluorinated polyvinyl chloride membrane and sealed with 5% skimmed milk for 2 h. Membranes were incubated overnight with primary antibodies against HSP70 (1:2000; ab2787, Abcam, USA), TSG101 (1:2000; 28283-1-AP, Proteintech, China), *NOX1* (1:1500; 17772-1-AP, Proteintech, China), and GAPDH (1:3000; ab8245; Abcam, China) at 4 °C, followed by secondary HRP-labeled goat anti-rabbit IgG (A0208, Beyotime, China) or HRP-labeled goat anti-mouse IgG (A0216, Beyotime, China). Proteins were observed using an enhanced chemiluminescence detection kit (Pierce Biotechnology, USA), with detection of GAPDH as the control.

### Cell counting kit-8 (CCK-8) assay

Cells (2 × 10^4^ cells/mL) were plated on a 96-well plate (100 μL/well) and incubated at 37 °C under 5% CO_2_ for 24 h with different concentrations of Ace. After 24 h of culture, CCK-8 solution was added to each well and cell viability was estimated by measuring absorbance at 450 nm using a microplate reader (VICTOR Nivo, Perkin Elmer, USA).

### Transwell assay

Cell invasion ability was evaluated with the Transwell chamber assay (8 μm, Corning Costar, USA). Cells were implanted into the upper compartment and the lower compartment filled with 20% serum. The filter was fixed and cultured for 48 h prior to staining with methanol and 0.1% crystal violet. A cotton swab was utilized to wipe the cells in the upper compartment. Cells below the filter were counted and images obtained under a microscope (IX83, OLYMPUS, JAPAN). All experiments were repeated three times.

### Wound healing assay

Aliquots of cells were inoculated on 6-well plates (1 × 10^6^/well). A single layer of cells was scratched with the tip of a pipette to make a notch in the dish. Images of the wound were captured at 0, 6, 24, and 48 h under a light microscope (ECLIPSE NI, Nikon, Japan). Wound closure was evaluated as follows: wound width (0 h)–wound width (6, 24, or 48 h)/wound width (0 h).

### ROS detection

Cells were incubated with H2DCFDA (HY-D0940, MCE, USA) for 30 min. After washing with cold PBS three times, images of cells were acquired via confocal scanning laser microscopy (ECLIPSE Ts2, Nikon, Japan).

### In vivo experiments

HeLa, HeLa-NC, and HeLa-OE cells were collected and the cell density adjusted to 1 × 10^8^/mL. Mice were captured and left forelimb was fixed. The left underarm skin was wiped with an alcohol cotton ball. A syringe was subcutaneously inserted into the left armpit in parallel and the tip moved from side to side twice to inject 100 µL of the cell suspension. Subsequently, the syringe was slowly removed and mice transferred into cages to continue feeding. The drug was administered 10 days later and samples collected after 4 weeks of intervention. Mice were euthanized via spinal dislocation. All animal experiments were approved by the Ethics Committee of our hospital. The method of administration is depicted in Additional file 2: Table S3. GW4869 is an inhibitor of exosome biogenesis/release [13].

### Statistical analysis

Cell experiments were performed at least three times and animal experiments at least six times. Statistical analysis was conducted using SPSS 22.0 software and GraphPad Prism 7.0. Numerical data are expressed as the means ± SD. The Student’s t-test and one-way ANOVA were applied to compare differences between groups. Differences were considered statistically significant at *P* < 0.05.

## Results

### *NOX1* is highly expressed with a mutational frequency of < 3% in pan-carcinoma

**Fig. 1:**
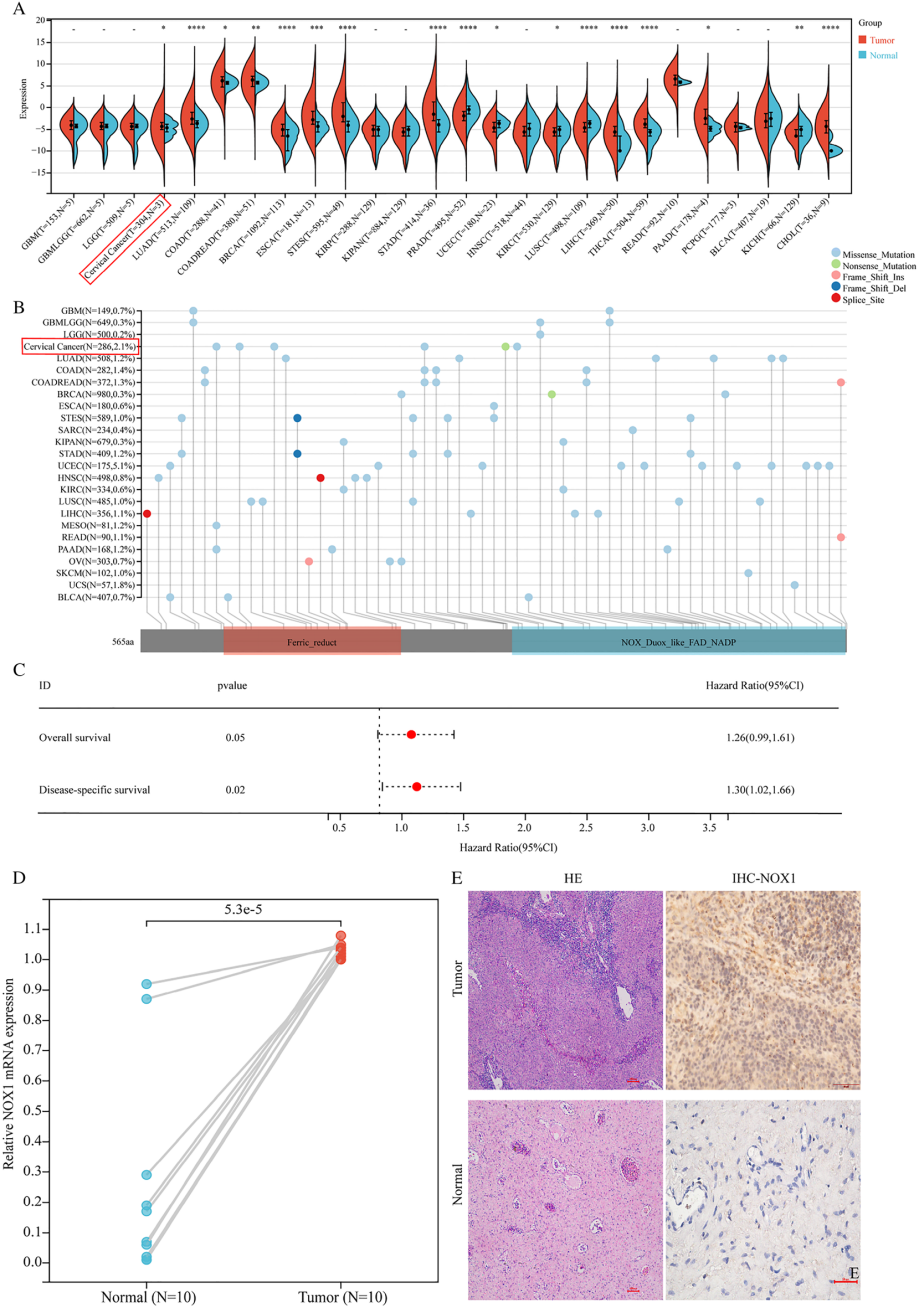
*NOX1* is highly expressed and maintained at a low mutational frequency in pan-carcinoma. **A** *NOX1* is highly expressed in tissues of many tumor types compared with adjacent normal tissues. **B** Low mutational frequency of *NOX1* in tissues of multiple tumor types. **C** Univariate analysis showing that *NOX1* is a potential risk factor for patient prognosis. **D** RT-PCR and **E** IHC detection of *NOX1* expression in paired adjacent normal and tumor tissues

Based on the TCGA database, we systematically evaluated the expression patterns and mutational frequency of *NOX1* in pan-carcinoma. Within 26 tumor types, *NOX1* was highly expressed in tumor compared to adjacent normal tissues (Fig. 1A). In addition, *NOX1* displayed low mutational frequency in 25 tumor tissues (Fig. 1B). In particular, *NOX1* was highly expressed in cervical cancer tissues relative to the non-tumor counterparts (Fig. 1A) with a mutational frequency of 2.1% (Fig. 1B). Univariate analysis revealed mRNA expression of *NOX1* as a risk factor for poor overall and disease-specific survival (Fig. 1C). We further evaluated the expression patterns of *NOX1* in cervical cancer. RT-PCR (Fig. 1D) and IHC (Fig. 1E) results consistently showed elevated expression of *NOX1* in cervical cancer tissues compared with adjacent normal tissues, prompting further investigation of the potential functions and mechanisms of action of *NOX1* in cervical cancer.

### Elevated expression of *NOX1* may be associated with infiltration of M2-type macrophages in cervical cancer tissues

**Fig. 2:**
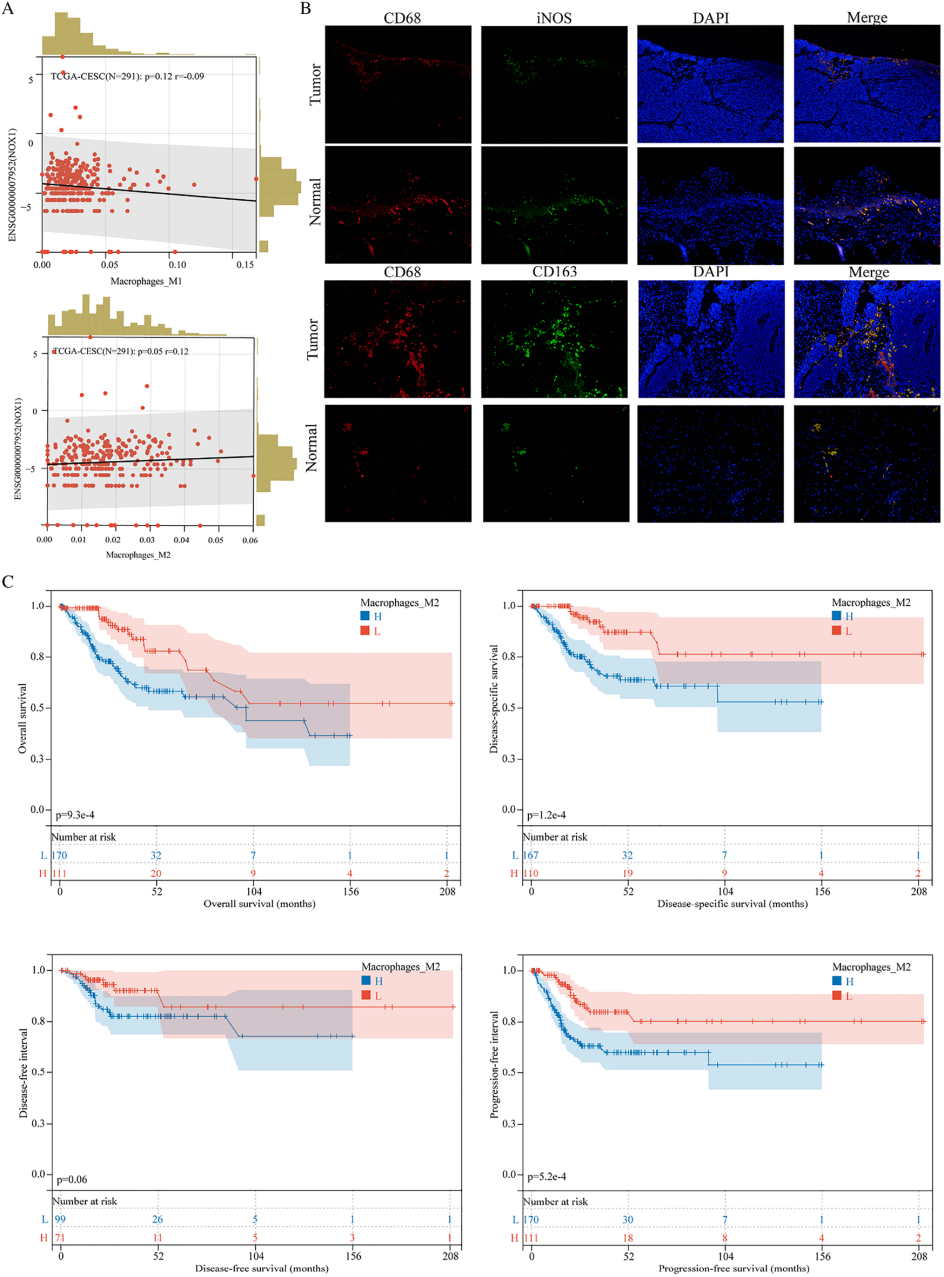
*NOX1* promotes infiltration of M2-type macrophages into cervical cancer tissues. **A** QUANTISEQ-based prediction of the correlation between *NOX1* expression and macrophages. **B** Fluorescence confocal microscopy detection of the infiltration of M1(CD68 + iNOS) and M2(CD68 + CD163) macrophages in adjacent normal tissues and tumor tissues (20x). **C** Kaplan–Meier survival analysis showing significant association of a higher proportion of M2 macrophages with poor prognosis. H, high, L, low. Scale bar, 100 µm for HE, 50 µm for IHC

Based on QUANTISEQ analysis, a significant positive correlation between *NOX1* expression and M2-type, but not M1-type macrophage infiltration was observed in cervical cancer tissues (Fig. 2A). Confocal fluorescence analyses showed that M1-type macrophages were substantially reduced, while M2-type macrophages were substantially increased in cervical cancer compared with adjacent normal tissues (Fig. 2B). In Kaplan–Meier survival analysis, a higher proportion of M2 macrophages was associated with poor prognosis of patients with cervical cancer (Fig. 2C). The collective results suggest that poor prognosis associated with the overexpression of *NOX1* is attributed to its activity in promoting M2 polarization of macrophages.

### ***NOX1*** promotes malignant features of cervical cancer cell by stimulating ROS production

**Fig. 3:**
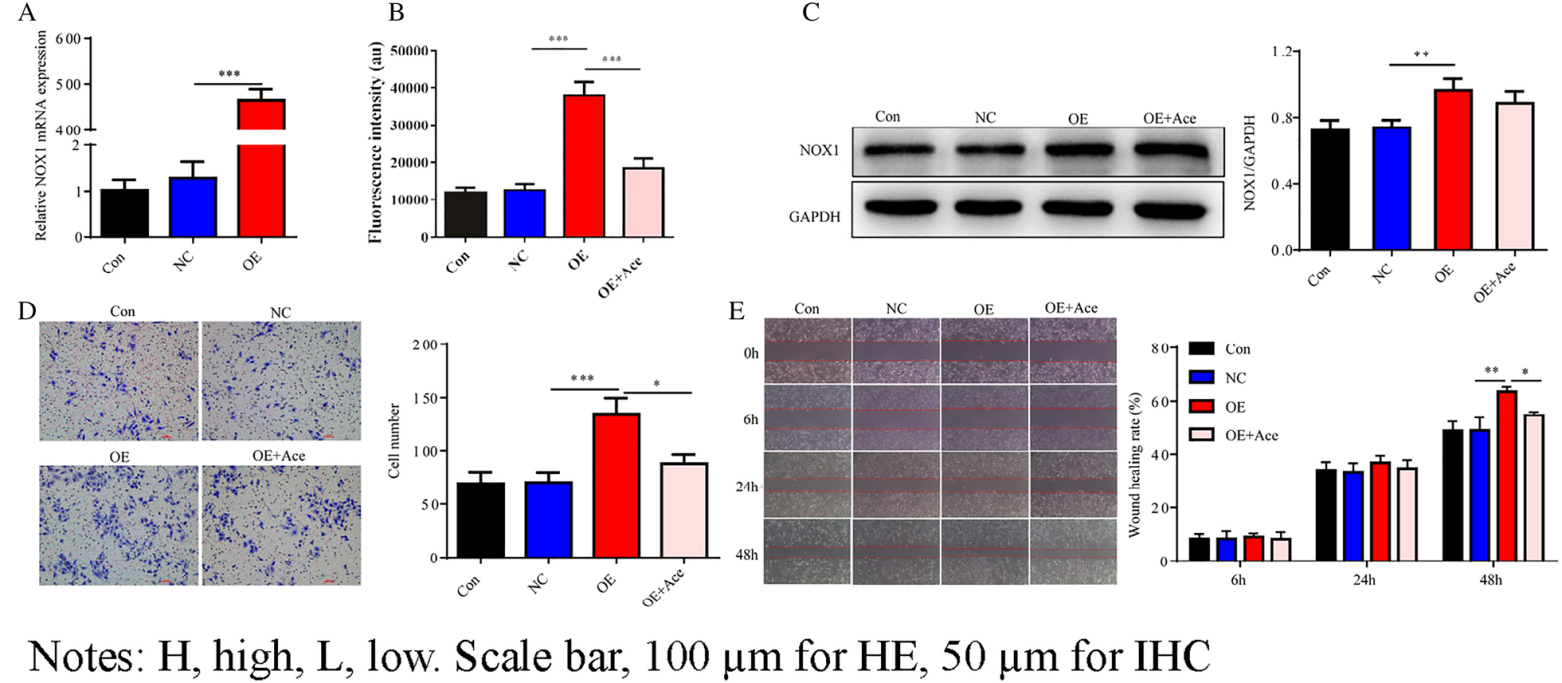
*NOX1* promotes malignant characteristics of HeLa cells by stimulating ROS production. **A** RT-PCR analysis of the effects of *NOX1*-overexpressing plasmids. **B** Detection of ROS levels for evaluation of *NOX1* overexpression and the inhibitory effect of Acetylcysteine. **C** WB analysis of *NOX1* expression in individual groups of cells. **D** Transwell and **E** scratch experiments for detecting the invasion and migration of HeLa cells in each group. Con, control; NC, negative control; OE, *NOX1* overexpression; Ace, Acetylcysteine. Scale bar, 50 µm

Plasmids overexpressing *NOX1* were generated and their transfection efficiencies examined using RT-PCR (Fig. 3A). The CCK-8 assay was used to determine the optimal concentrations of ROS inhibitors (Acetylcysteine, Ace). The experiments showed that 50 µM Ace induced a substantial reduction in proliferation of HeLa cells (Additional file 1: Fig. S1). Overexpression of *NOX1* led to a significant increase in the level of ROS. Treatment with the ROS inhibitor, Ace, induced a significant decrease in the ability of cells overexpressing *NOX1* to generate ROS (Fig. 3B). WB analysis disclosed higher expression of *NOX1* in the *NOX1*-OE compared with *NOX1*-NC group but no significant differences between the *NOX1*-OE and *NOX1*-OE + Ace groups (Fig. 3C). Notably, Transwell (Fig. 3D) and scratch (Fig. 3E) experiments demonstrated that invasion and migration abilities of HeLa cell were substantially reduced in the *NOX1*-OE + Ace compared to *NOX1*-OE group. Our findings clearly suggest that *NOX1* promotes malignant features of cervical cancer cells via stimulation of ROS production.

### Exosomal *NOX1* promotes M2 polarization of macrophages by stimulating ROS production

**Fig. 4:**
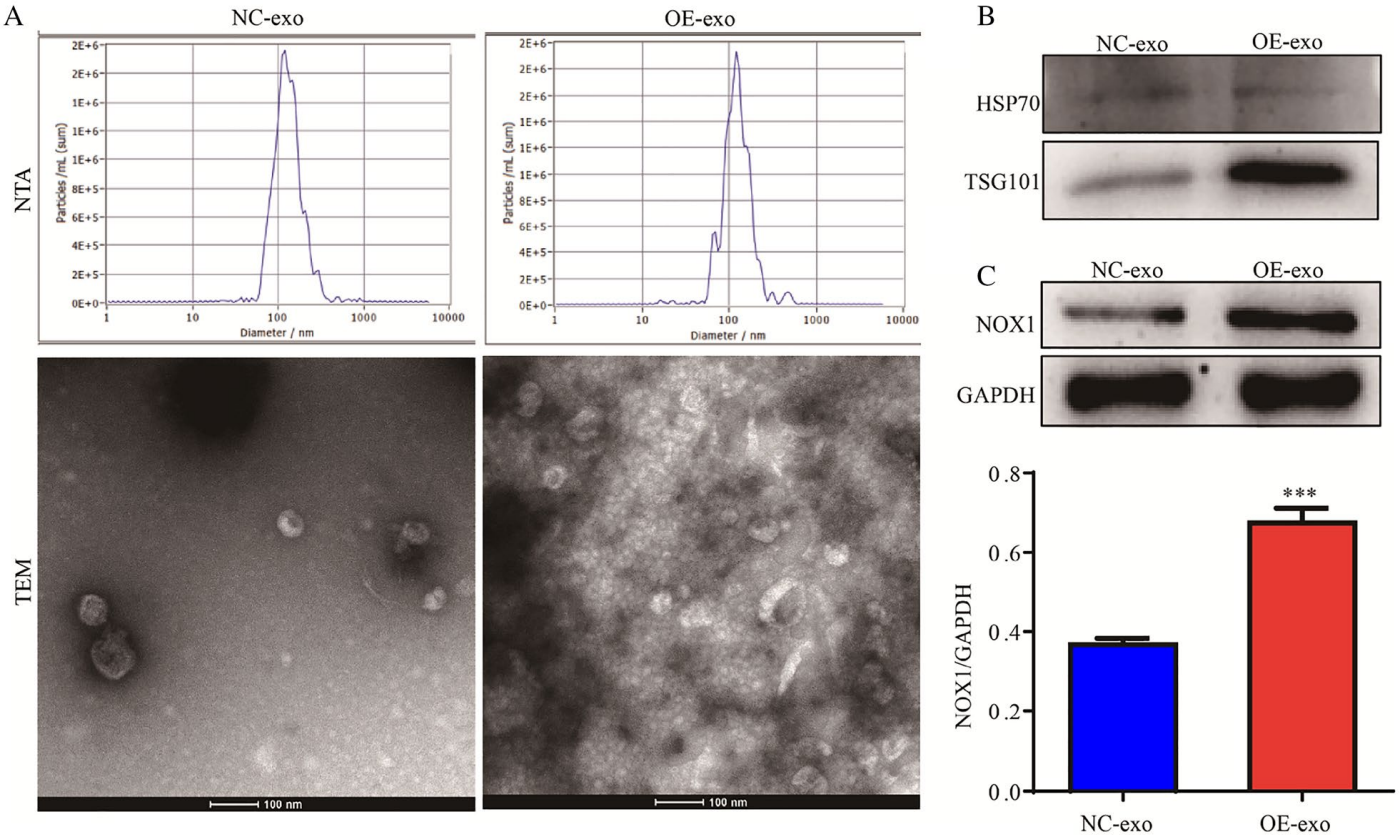
Identification of exosomes. **A** NTA and TEM analysis of exosome particle size and shape, respectively. **B** WB analysis of exosomal marker proteins. **C** WB analysis of *NOX1* expression in exosomes. Notes: exo, exosome

We utilized nanoparticle tracking analysis (NTA) to detect particle size and transmission electron microscopy (TEM) analysis to determine the morphology of NC-exo and OE-exo (Fig. 4A). WB was employed to detect the exosomal markers HSP70 and TSG101 (Fig. 4B) and further revealed substantially higher expression of *NOX1* in the OE-exo compared to NC-exo group (Fig. 4C).

**Fig. 5:**
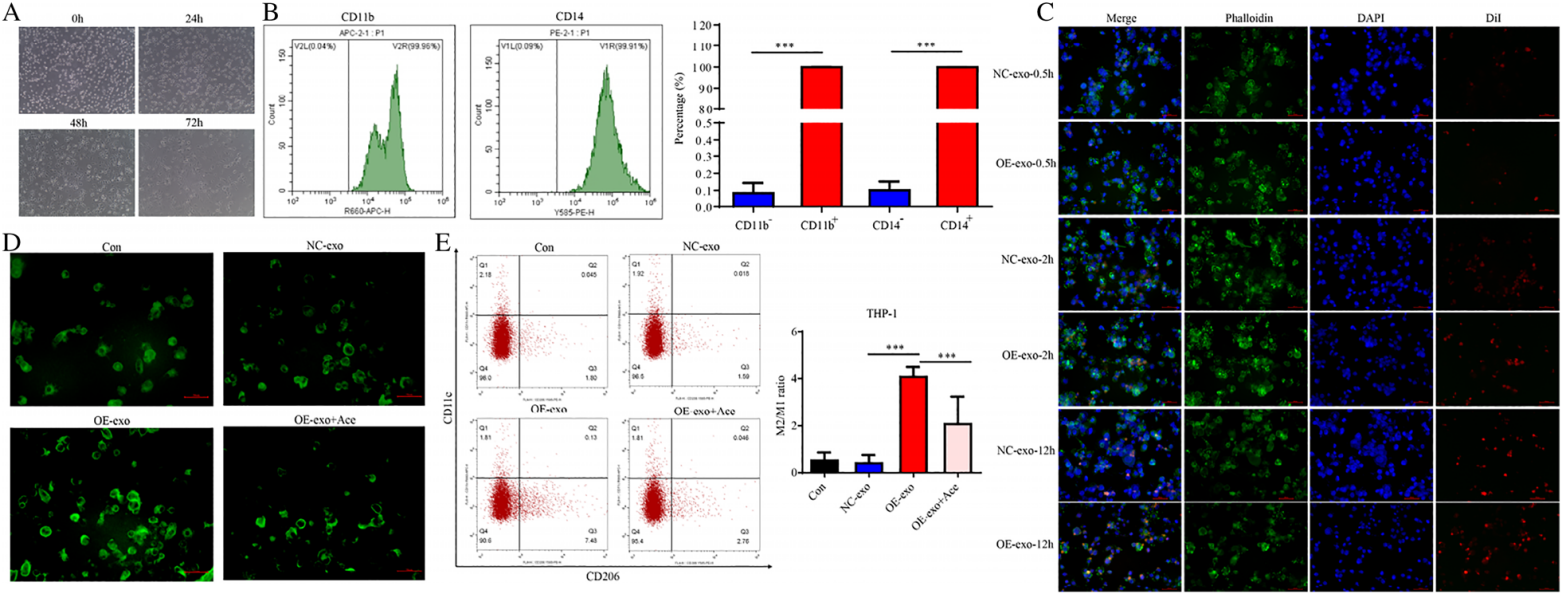
*NOX1* promotes polarization of M0- to M2-type macrophages by stimulating ROS production. **A** Electron microscopy analysis of cell morphology. **B** Flow cytometry detection of CD11b and CD14 expression. **C** Fluorescence confocal microscopy for evaluation of exosomal uptake in M0 macrophages. **D** Fluorescence microscopy for evaluation of ROS production. **E** Flow cytometry detection of polarization of macrophages. Scale bar, 50 µm. Con, control; NC, negative control; OE, *NOX1* overexpression; Ace, Acetylcysteine; exo, exosome

Electron microscopy (Fig. 5A) and flow cytometry (Fig. 5B) results showed that PMA successfully induced the conversion of THP-1 to M0 macrophages. Data obtained from fluorescence confocal microscopy analysis showed that M0 macrophages ingest exosomes secreted by HeLa cells (Fig. 5C). The ROS concentration in each group of cells was further determined using H2DCFDA (Fig. 5D). Flow cytometry experiments showed that OE-exo promoted M2-type polarization of M0 macrophages compared to the NC-exo group. However, polarization of M0- to M2-type macrophages was substantially reduced in OE-exo + Ace relative to the OE-exo group (Fig. 5E). The collective results suggest that exosomal *NOX1* promotes M2 polarization of macrophages through stimulating ROS production.

### Exosomal ***NOX1*** enhances progression of cervical cancer and M2 polarization of macrophages in vivo by stimulating ROS production

**Fig. 6:**
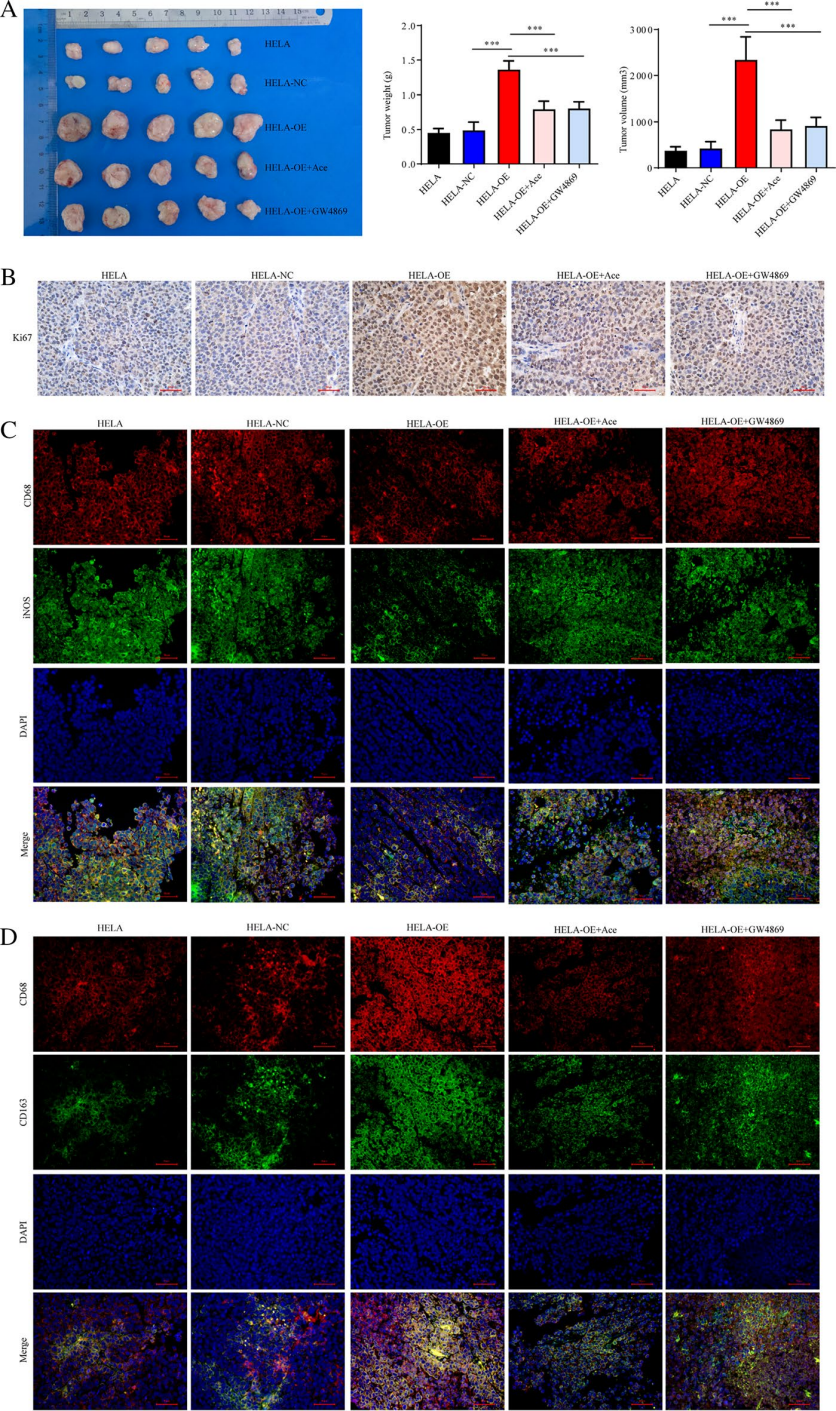
Exosomal *NOX1* promotes tumor growth and M2 macrophage infiltration in vivo by stimulating ROS production. **A** Experiments on tumor-bearing nude mice. **B** IHC analysis of Ki67 expression. **C** Fluorescence confocal microscopy-based detection of iNOS and CD68 expression. **D** Fluorescence confocal microscopy-based detection of CD68 and CD163 expression. Scale bar, 50 µm; Con, control; NC, negative control; OE, *NOX1* overexpression; Ace, Acetylcysteine; exo, exosome; GW4869, inhibitor of exosome biogenesis/release

Compared with the HeLa-NC treatment group, the weight and volume of tumor tissues in the HeLa-OE group were significantly increased. However, relative to the HeLa-OE group, the weight and volume of tumor tissues in the HeLa-OE + Ace and HeLa-OE + GW4869 treatment groups were markedly decreased (Fig. 6A). IHC results showed that expression of Ki67 in tumor tissues was substantially increased in HeLa-OE compared with the HeLa-NC group. However, Ki67 expression in tumor tissues was significantly reduced in HeLa-OE + Ace and HeLa-OE + GW4869 groups relative to the HeLa-OE group (Fig. 6B). Fluorescence confocal microscopy revealed a significant increase in the number of M2-type macrophages and, conversely, decrease in M1-type macrophages in the HeLa-OE group compared with the HeLa-NC group. Relative to the HeLa-NC group, the M1-type macrophage content was substantially increased, while that of M2-type macrophages was decreased in HeLa-OE + Ace and HeLa-OE + GW4869 treatment groups (Fig. 6C, D). Based on these results, we propose that exosomal *NOX1* promotes tumor growth and M2 macrophage infiltration by stimulating ROS production.

## Discussion

Bioinformatics analysis revealed a < 6% mutational frequency of *NOX1* across pan-cancer tumor types. However, expression of *NOX1* was significantly higher in cancer tissues relative to the corresponding adjacent normal counterparts, including cervical cancer. This differential expression of *NOX1* may be associated with tumor occurrence and progression.

RT-PCR and IHC analyses confirmed the significant overexpression of *NOX1* in cervical cancer compared with adjacent normal tissues. Similar results based on tissue microarray immunohistochemical staining were reported by Jiang et al. [6]. Data from Transwell and scratch experiments showed a strong association of *NOX1* overexpression with enhanced invasion and migration of HeLa cells, consistent with earlier findings by Jiang et al. [6] that *NOX1* promotes the invasion and migration of SiHa and HCC94 cell lines. Furthermore, overexpression of *NOX1* promoted HeLa cell growth in vivo, supporting its carcinogenic role in cervical cancer.

Macrophages are one of the major cell types in the innate immune system [14]. Macrophages in the tumor microenvironment (designated TAMs) account for 30–50% of cells in the tumor microenvironment. TAMs are involved in invasion, migration, and several other biological processes of tumors [15]. Depending on the phenotype, TAMs are divided into M1 and M2 macrophages [16]. M1 macrophages display the characteristic property of releasing proinflammatory cytokines and are considered anti-tumor factors. However, M2-type macrophages have low antigen presenting ability and low anti-tumor activity, which are beneficial for tumor growth and invasion [17]. Based on QUANTISEQ analysis, we observed an almost significant positive correlation between *NOX1* expression and M2 macrophages. High expression of *NOX1* is reported to be associated with poor prognosis of cervical cancer patients [7, 8]. Consistently, our results showed that the higher proportion of M2 macrophages was associated with poor prognosis of patients with cervical cancer. Both in vitro and in vivo experiments collectively demonstrate that overexpression of *NOX1* promotes the polarization of macrophages to M2 type, suggesting a carcinogenic role of *NOX1* via this pathway. Combined with the findings of Jiang et al. [7, 8], we infer from these experiments that *NOX1* promotes M2 polarization of macrophages, which contributes to poor prognosis of patients with cervical cancer.

Exosomes play an important role in intercellular communication among not only cancer cells but also between cancer cells and immune cells [18]. Diverse roles of exosomes in cervical cancer have been reported, including exosomal lncRNA- or microRNA-based promotion of cell proliferation [19], angiogenesis [20], and immune escape [21]. In the present study, exosomal *NOX1* promoted tumor growth and M2 macrophage infiltration in vivo by stimulating ROS production. In both in vitro and in vivo assays, compared with the *NOX1* overexpression group, treatment with the exosome inhibitor, GW4869, induced a significant decrease in *NOX1*-mediated invasion, migration, and growth of HeLa cells and inhibited M2 polarization of macrophages. These results indicate that HeLa cells promote M2 polarization of macrophages by secreting *NOX1*-carrying exosomes, in turn, stimulating M2 macrophage-mediated carcinogenesis. The collective findings provide a valuable insight into the potential mechanisms by which oncogenic *NOX1* promotes cervical cancer progression.

The present study had some limitations that should be taken into consideration. The main research results were based on in vitro cell line experiments, and further in vivo animal experiments and clinical sample verification are needed to elucidate the specific mechanisms by which exosome *NOX1*-derived ROS induce M2 macrophage polarization.

In summary, exosomal *NOX1* promotes tumor-associated macrophage M2 polarization-mediated cancer progression through stimulating ROS production in cervical cancer.

### Supplementary Information

**Additional file 1: Figure S1.** CCK-8 was used to detect the optimum concentration of ACE.

**Additional file 2: Table S1.** Abbreviation. **Table S2.** The Pathological diagnosis of clinical samples. **Table S3.** All primers used in this experiment.

## Data Availability

The data sets used or analyzed during the current study are available from the corresponding author on reasonable request.
