# Supplementary figures and images for: Exosomal NOX1 promotes tumor-associated macrophage M2 polarization-mediated cancer progression by stimulating ROS production in cervical cancer: a preliminary study
Source: Eur J Med Res. 2023 Sep 7;28:323. doi: 10.1186/s40001-023-01246-9 (PMC10483767; doi:10.1186/s40001-023-01246-9)

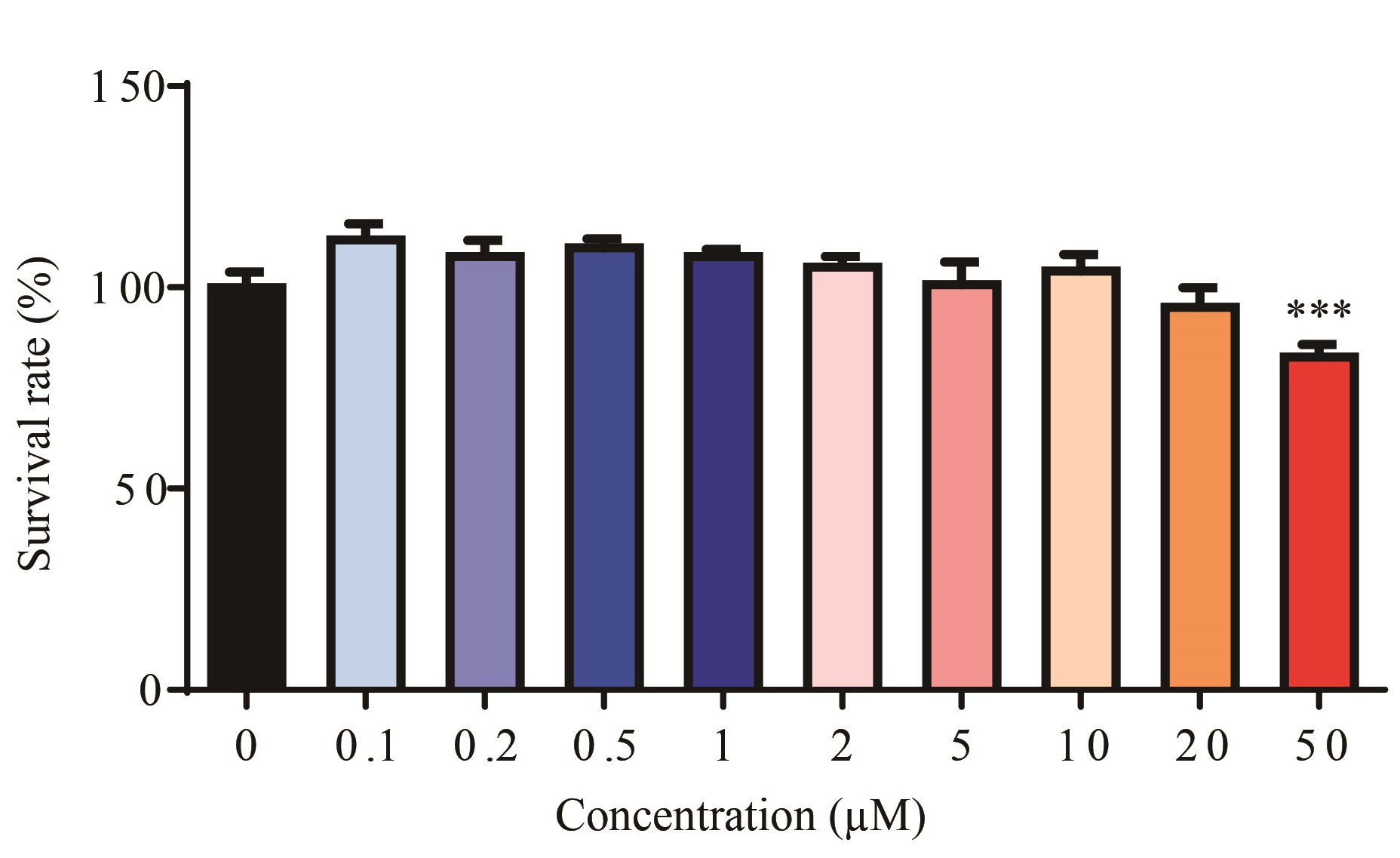

Supplement: Supplementary file 1 — Additional file 1: Figure S1. CCK-8 was used to detect the optimum concentration of ACE. [file 40001_2023_1246_MOESM1_ESM.tif]
