# Supplementary material for: Exosomal NOX1 promotes tumor-associated macrophage M2 polarization-mediated cancer progression by stimulating ROS production in cervical cancer: a preliminary study
Source: Eur J Med Res. 2023 Sep 7;28:323. doi: 10.1186/s40001-023-01246-9 (PMC10483767; doi:10.1186/s40001-023-01246-9)
Supplement: Supplementary file 2 — Additional file 2: Table S1. Abbreviation. Table S2. The Pathological diagnosis of clinical samples. Table S3. All primers used in this experiment. [file 40001_2023_1246_MOESM2_ESM.docx]

Additional file

Table S1. Abbreviation

| Cohort | Full name |
| --- | --- |
| TCGA-ACC | Adrenocortical carcinoma |
| TCGA-BLCA | Bladder Urothelial Carcinoma |
| TCGA-BRCA | Breast invasive carcinoma |
| TCGA-CESC | Cervical squamous cell carcinoma and endocervical adenocarcinoma |
| TCGA-CHOL | Cholangiocarcinoma |
| TCGA-COAD | Colon adenocarcinoma |
| TCGA-READ | Rectum adenocarcinoma Esophageal carcinoma |
| TCGA-DLBC | Lymphoid Neoplasm Diffuse Large B-cell Lymphoma |
| TCGA-ESCA | Esophageal carcinoma |
| TCGA-FPPP | FFPE Pilot Phase II |
| TCGA-GBM | Glioblastoma multiforme |
| TCGA-LGG | Glioma |
| TCGA-HNSC | Head and Neck squamous cell carcinoma |
| TCGA-KICH | Kidney Chromophobe |
| TCGA-KIPAN | Pan-kidney cohort (KICH+KIRC+KIRP) |
| TCGA-KIRC | Kidney renal clear cell carcinoma |
| TCGA-KIRP | Kidney renal papillary cell carcinoma |
| TCGA-LAML | Acute Myeloid Leukemia |
| TCGA-LGG | Brain Lower Grade Glioma |
| TCGA-LIHC | Liver hepatocellular carcinoma |
| TCGA-LUAD | Lung adenocarcinoma |
| TCGA-LUSC | Lung squamous cell carcinoma |
| TCGA-MESO | Mesothelioma |
| TCGA-OV | Ovarian serous cystadenocarcinoma |
| TCGA-PAAD | Pancreatic adenocarcinoma |
| TCGA-PCPG | Pheochromocytoma and Paraganglioma |
| TCGA-PRAD | Prostate adenocarcinoma |
| TCGA-READ | Rectum adenocarcinoma |
| TCGA-SARC | Sarcoma |
| TCGA-STAD | Stomach adenocarcinoma |
| TCGA-SKCM | Skin Cutaneous Melanoma |
| TCGA-STES | Stomach and Esophageal carcinoma |
| TCGA-TGCT | Testicular Germ Cell Tumors |
| TCGA-THCA | Thyroid carcinoma |
| TCGA-THYM | Thymoma |
| TCGA-UCEC | Uterine Corpus Endometrial Carcinoma |
| TCGA-UCS | Uterine Carcinosarcoma |
| TCGA-UVM | Uveal Melanoma |
| TARGET-OS | Osteosarcoma |
| TARGET-ALL | Acute Lymphoblastic Leukemia |
| TARGET-NB | Neuroblastoma |
| TARGET-WT | High-Risk Wilms Tumor |

Table S2：The Pathological diagnosis of clinical samples

| case | age | Pathological diagnosis |
| --- | --- | --- |
| 1 | 54 | adenocarcinoma |
| 2 | 69 | adenocarcinoma |
| 3 | 34 | adenocarcinoma |
| 4 | 77 | Squamous Cell Carcinoma |
| 5 | 60 | Squamous Cell Carcinoma |
| 6 | 67 | Squamous Cell Carcinoma |
| 7 | 39 | Squamous Cell Carcinoma |
| 8 | 46 | Squamous Cell Carcinoma |
| 9 | 67 | Squamous Cell Carcinoma |
| 10 | 39 | Squamous Cell Carcinoma |

Table S3. All primers used in this experiment

| Gene | Forward | Reverse |
| --- | --- | --- |
| NOX1 | CAAGGCCACTGACATCGTGA | CAAAGTCCGAGGGCCACATA |
| GAPDH | TCACTTCAACAGCGACACCCA | CACCCTGTTGCTGTAGCCAAA |
